# Genotype × environment interaction and stability of grain micronutrients in wheat under organic and conventional systems: AMMI and GGE analysis

**DOI:** 10.3389/fpls.2026.1785559

**Published:** 2026-04-13

**Authors:** Mohamad I. Motawei, Nasser S. Al-Ghumaiz, Soleman M. Al-Otayk, Essam M Abd-Elmoniem, Ahmed M. Aggag

**Affiliations:** 1 Department of Plant Production, College of Agriculture and Food, Qassim University, Buraydah, Qassim, Saudi Arabia; 2 Department of Environment and Natural Resources, College of Agriculture and Food, Qassim University, Buraydah, Saudi Arabia

**Keywords:** AMMI, crop genetic diversity, genotype × environment interaction, GGE biplot, micronutrient content, stability analysis, wheat

## Abstract

Improving the micronutrient content of wheat grains remains an important objective for addressing micronutrient deficiencies in human diets. This study evaluated the performance and stability of seven wheat (*Triticum aestivum* L.) genotypes for grain iron (Fe), zinc (Zn), manganese (Mn), copper (Cu), and selenium (Se) concentrations across six environments, defined by three growing seasons under organic and conventional fertilization systems. Genotype, environment, and genotype × environment (G×E) effects were examined using Additive Main Effects and Multiplicative Interaction (AMMI) analysis and Genotype plus Genotype-by-Environment (GGE) biplot methods. Combined ANOVA demonstrated highly significant (P ≤ 0.01) influences of both G and E on all micronutrients, whereas GEI effects were dependent on the nutrient and significant for Mn, Zn, and Se. The initial two AMMI interaction principal component axes (IPCA1 and IPCA2) accounted for 99.63%, 90.59%, 78.57%, 93.05%, and 90.16% of the GEI sum of squares for Fe, Mn, Zn, Cu, and Se, respectively. Genotype stability was measured using the AMMI Stability Value (ASV), a composite measure based on IPCA scores, along with the Genotype Selection Index (GSI), which combines average performance and stability rankings. Patterns of adaptation specific to systems were identified across different fertilization regimes. The Local genotype concentrations of Fe (91.27 ppm), Mn (44.47 ppm), and Zn (50.29 ppm) rank as one of the top genotypes in both organic and conventional systems. IC8 consistently demonstrated the highest Se concentration (1316.6 ppb) and retained stable positions across both organic and conventional systems, suggesting wide adaptability. Conversely, P5 and IC17 demonstrated enhanced Cu and Zn performance mainly under conventional conditions, while Sids12 exhibited decreased stability in organic conditions. GGE biplot analysis further categorized test environments into system-defined mega-environments, demonstrating differing genotype adaptation between organic and conventional fertilization methods. In general, Local and IC8 demonstrated significant micronutrient accumulation alongside low sensitivity to GEI (low ASV and GSI), suggesting their potential as parental lines for developing wheat cultivars with broad or specific adaptation in biofortification initiatives aimed at low-input (organic) and high-input (inorganic) production systems.

## 1 Introduction

Wheat (*Triticum aestivum* L.) serves as a key staple crop that underpins global food and nutritional security, especially in areas where cereal-rich diets form the main source of daily calories. Nevertheless, wheat grain generally has inadequate levels of vital micronutrients such as iron (Fe), zinc (Zn), and selenium (Se), which contribute to the common occurrence of micronutrient malnutrition, often referred to as hidden hunger (Hefferon, 2015; Bouis and Saltzman, 2017; Bailey et al., 2015). Increasing the density of micronutrients in grains via genetic biofortification has become a key goal in modern wheat breeding initiatives focused on sustainably enhancing dietary quality (Cakmak and Kutman, 2018).

Enhancing the micronutrient concentration in grains through genetics poses a significant breeding challenge owing to the quantitative nature of these traits and their strong sensitivity to environmental fluctuations. The accumulation of minerals in grains is controlled by intricate physiological and genetic mechanisms that are significantly affected by soil characteristics, climate conditions, and fertilization methods. Consequently, genotype × environment interaction (GEI) significantly impacts micronutrient expression in various production settings, frequently resulting in variable genotype rankings and decreased selection efficiency (Meena et al., 2025). Earlier research has shown that environmental factors often contribute significantly to the phenotypic variation in Zn, Mn, Cu, and Se accumulation, while Fe content might display comparatively more genetic influence (Borg et al., 2012). This variability highlights the importance of including stability analysis in breeding approaches to improve micronutrient content.

Multi-environment trials (METs) are commonly conducted to assess genotype performance and suitability across various environmental settings. Analytical methods like Additive Main Effects and Multiplicative Interaction (AMMI) and Genotype plus Genotype-by-Environment Interaction (GGE) biplot models are well-known for their ability to divide phenotypic variation into genotype, environment, and interaction factors, which facilitates the detection of genotypes that are stable and have broad adaptability (Yan and Kang, 2020). In wheat, AMMI analysis has been effectively used to evaluate the stability of genotypes concerning agronomic traits; for instance, Al-Ghumaiz et al. (2025) found that the wheat genotype IC8 attained the highest average grain yield (1.868 t ha^-^¹) and the lowest AMMI Stability Value (ASV = 0.474), suggesting excellent yield stability throughout the environments tested.

Nonetheless, the use of AMMI and GGE models in wheat has mainly concentrated on grain yield stability, with relatively little focus on micronutrient traits, especially under differing fertilization conditions. This constraint highlights a significant void in present biofortification-focused breeding studies. Earlier AMMI- and GGE-focused studies have assessed genotype performance under consistent agronomic practices, ignoring the effects of fertilization system-induced environmental variability on G×E interaction for micronutrient accumulation (Crossa et al., 1990).

To date, limited attention has been given to evaluating the stability of grain micronutrient accumulation using these approaches, particularly under contrasting fertilization systems such as organic and conventional management (Crossa et al., 1990). This represents a critical knowledge gap for biofortification-oriented breeding, as nutrient availability, uptake efficiency, and genotype responsiveness may differ substantially between low-input and high-input production environments. Considering that nutrient availability, uptake efficiency, and soil–plant nutrient interactions vary significantly between organic and conventional fertilization systems, genotypes chosen for enhanced micronutrient content in one production system may not exhibit consistent performance in another (Mullualem et al., 2024).

Thus, integrating stability analysis of micronutrient characteristics across differing management systems is crucial for enhancing selection precision in biofortification breeding programs. In this setting, the joint use of AMMI and GGE biplot techniques offers a solid framework for measuring G×E interaction impacts and evaluating genotype adaptability for micronutrient accumulation in various fertilization conditions (Yan and Kang, 2020). In this context, the present study advances beyond previous AMMI/GGE applications by integrating multi-nutrient stability analysis with management-system-based environmental stratification. Specifically, this study evaluates genotype adaptability and GEI sensitivity for grain Fe, Zn, Mn, Cu, and Se concentrations across environments defined not only by seasonal variability but also by fertilization regime. This approach enables the identification of wheat genotypes combining high micronutrient accumulation with low interaction effects across contrasting input systems. Therefore, the objectives of this study were to:

(i) quantify the relative contributions of genotype, fertilization system, and genotype × environment interaction to variation in grain micronutrient content using AMMI analysis; (ii) assess genotype stability for micronutrient traits using AMMI Stability Value (ASV) in conjunction with GGE biplot analysis; and (iii) identify wheat genotypes exhibiting wide or system-specific adaptation for micronutrient accumulation under organic and conventional fertilization systems for use in biofortification-oriented breeding programs.

## 2 Materials and methods

### 2.1 Experimental design and treatments

Field trials were carried out for three successive growing seasons from 2019 to 2021 at the Agricultural Research and Experimental Station of Qassim University, located in Buraydah, Saudi Arabia (26°18′28″N, 43°46′00″E). The location features sandy texture, low salinity (EC = 1.5 dSm^-^¹), minimal organic matter (0.4%), and an alkaline pH of 8.1. During the study, the irrigation water utilized had an EC of 1.7 dSm^-^¹ and a pH level of 7.8.

The research assessed the effectiveness of seven wheat (*Triticum aestivum* L.) genotypes (**Table 1**) under two types of fertilization: organic (O) and inorganic (N). Consequently, the factors involved in the experiment were

**Table 1:** Seven wheat genotypes used in this study.

Genotype name	Source
Yocora Rojo (YR)^†^	USA
LOCAL^‡^	KSA
P3 (AUS-030851)	Australia
P5 (AUS-030852)	Australia
IC8 (Line-2-ICARDA-1^st^ RDRN0607)	ICARDA^§^
IC17 (Line-56 ICARDA-1^st^ RDRN0607)	ICARDA
Sids_12	Egypt

^†^Yocora Rojo (YR): commercial genotype commonly cultivated in Saudi Arabia. ‡ Local genotype (Sama).

- Factor 1 (Genotypes): Seven genotypes of wheat (*Triticum aestivum* L.).

- Factor 2 (Fertilization): Two systems—organic (O) and inorganic (N).

Experiments were arranged in a randomized complete block design (RCBD) consisting of three blocks. Every block included 14 plots (7 genotypes × 2 fertilization systems). Every plot area was 3 m² (1.5 m × 2.0 m) and consisted of 8 rows that were 25 cm apart. Wheat seeds were planted at a rate of 45 kg ha^-^¹. The sowing dates for the three seasons were December 2019, November 30, 2020, and December 2021.

### 2.2 Fertilizer application, environmental and soil conditions

**Figure 1:**
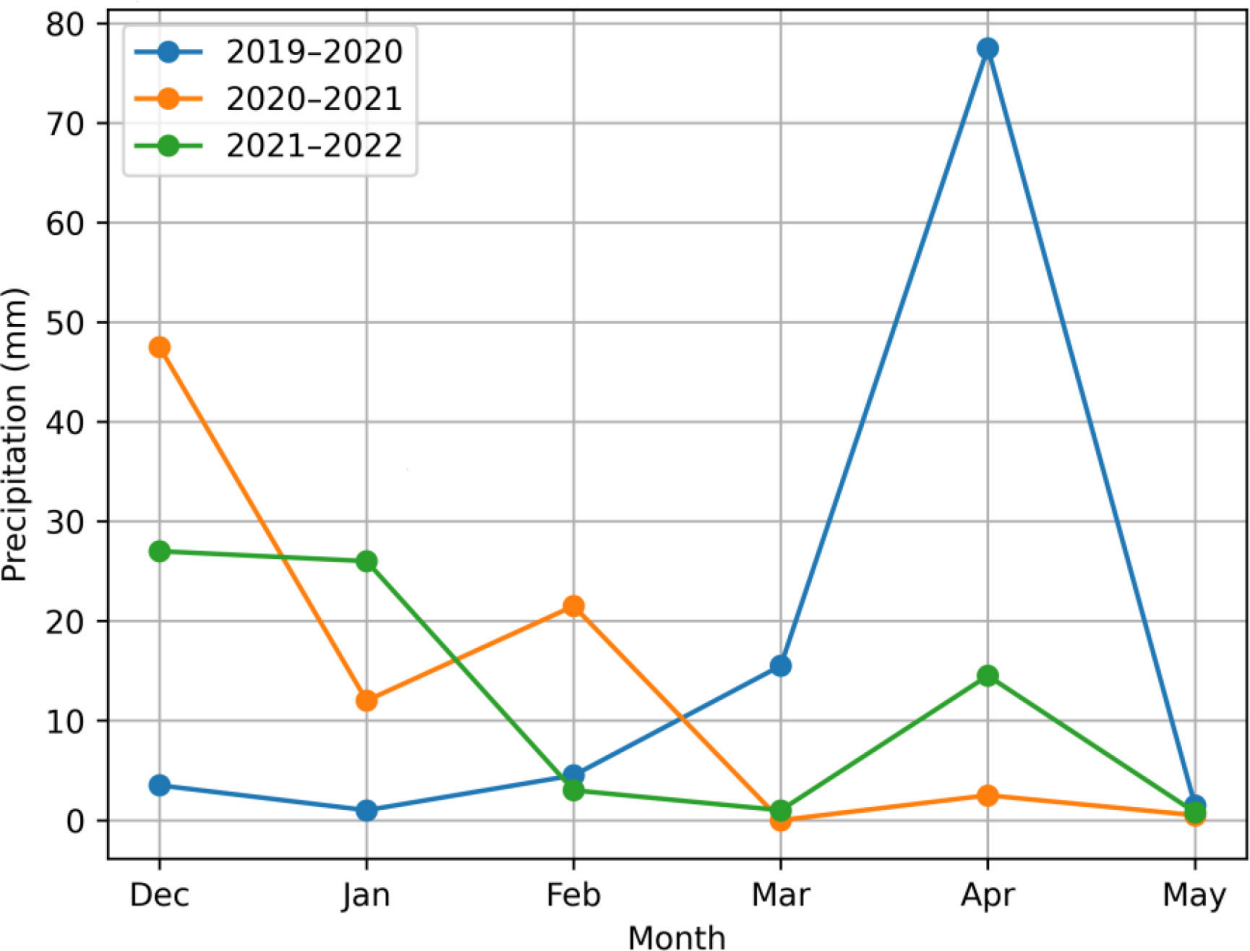
Three-year average (2019–2021) of monthly precipitation (December–May) in the Qassim region.

The application of fertilizer followed the recommendations based on soil testing. In the inorganic treatment, urea, diammonium phosphate (DAP), and potassium sulfate were used at rates of 124 kg N ha^-^¹, 92 kg P_2_O_5_ ha^-^¹, and 57 kg K_2_O ha^-^¹, respectively. For the organic treatment, well-composted cow manure was applied at a rate of 10 t ha^-^¹ one month before sowing. The organic amendment was evaluated using the methodology of Sparks et al. (2020), showing an N:P: K ratio of 0.5:0.21:0.5. **Figure 1** presents monthly precipitation (December–May) for the study period (2019–2021). **Table 2** presents a summary of environmental conditions over various years. The physical and chemical characteristics of the soil texture (at 0–25 cm in depth), pH, EC, organic matter, total N, available P, K, sand, silt, and clay (**Table 3**) were measured before experimentation according to Page et al. (1982).

**Table 2:** Environmental conditions* across different years and fertilizations [organic (O) and inorganic (N)] based on precipitation and temperature variables.

Number	Environment	Precipitation (mm)	T_max_ (°C)	T_min_ (°C)
1	2019-N	146.5	26.64	14.01
2	2019-O	146.5	26.64	14.01
3	2020-N	113.5	28.19	14.84
4	2020-O	113.5	28.19	14.84
5	2021-N	96.6	28.16	15.07
6	2021-O	96.6	28.16	15.07

*Terra Climate dataset, Climatology Lab, available at: http://www.climatologylab.org/terraclimate.html.

**Table 3:** Soil chemical and physical analyses for the two experiment sites.

Site	Chemical analysis						Particle size distribution (%)		
	K (ppm)	P (ppm)	N (ppm)	OM** (%)	pH	EC (dS.m^-1^)	Clay	Silt	Sand
Inorganic site*	34	33.1	15.7	0.4	8.1	1.3	0.9	4.2	94.9
Organic site*	36.5	22.1	52.5	0.4	7.9	1.7	1.0	4.5	94.5

*Qassim University Agricultural Research Station.

**OM, organic matter.

### 2.3 Micronutrient analysis

For the determination of Se, Fe, Mn, Cu, and Zn in the wheat grain, samples were rinsed with deionized water and dried at 100 °C for 25 min and then at 70 °C for 48 hr. All samples were sieved to > 0.15 and< 0.5 mm with a stainless-steel mill. One-gram quantities of each plant sample were digested with a mixture containing concentrated HNO_3_, HCIO_4_, and H_2_SO_4_ (7:2:1) (Page et al., 1982), and the Se, Fe, Mn, Cu, and Zn content were measured with ICP-OES (Model iCAP 7400 Duo, serial IC 74DC144208, assembled in China).

### 2.4 Statistical analysis

#### 2.4.1 AMMI analysis

The AMMI model (Gauch, 1992, 2013) (Equation 1) was utilized to assess the yield stability of the superior spring wheat genotypes with organic and inorganic fertilization. Principal component analysis (PCA) was employed to assess the multiplicative impacts of Genotype × Environment Interaction (GEI) following the initial fitting of the additive effects by the AMMI model for the main factors of genotypes (G) and environment (E). Biplot graphs were utilized to present the AMMI results. According to Nowosad et al. (2016), the AMMI model can be expressed through the following formula:

(1) $$Y_{ge}=\;\mu +\;\alpha_{g}+\;\beta_{e}+\;\sum_{n=1}^{N}\lambda_{n}\gamma_{gn}\delta_{en\;}+\;Q_{ge}$$

where N represents the number of PCA axes retained in the modified model; λ_n_ indicates the eigenvalue of the PCA axis, n; γ_gn_ denotes the genotype score for the PCA axis, n; δ_en_ is the score eigenvector corresponding to the PCA axis, n; Y_ge_ signifies the trait mean of a genotype g in environment e; μ is the overall mean; α_g_ is the deviation of the mean genotype; β_e_ is the deviation of the mean environment; and Q_ge_ accounts for the residual, which includes AMMI noise and combined experimental error. The “AMMI” function from the GenStat statistical software, version 19 (GenStat, 2019), was utilized to perform AMMI analysis.

#### 2.4.2 AMMI stability value

The stability of the genotypes was assessed using the AMMI stability value (ASV) coefficient (Equation 2) according to Purchase et al. (2000) as (Equation 2):

(2) $$ASV=\sqrt{{\left(\frac{SS_{IPCA1}}{SS_{IPCA2}}\;\left(IPCA1\right)\right)}^{2}\;+\;{\left(IPCA2\right)}^{2}}$$

where SSIPCA1/SSIPCA2 is the ratio between the sum of squares from the first and second interaction principal component axis, and IPCA1 and IPCA2 are the genotypic scores of these components in the AMMI model. The genotype with the lowest value of this statistic would be more stable. The more consistent the genotype in the examined conditions, the smaller the ASV. Every genotype received a genotype selection index (GSI), calculated as the sum of the ASV and yield stability index (YSI) ranking positions (Farshadfar, 2008).

#### 2.4.3 GGE biplot analysis

AGGE (Genotype + Genotype-by-Environment) biplot was created utilizing principal component analysis, where the genotype scores were multiplied by the respective environmental scores to generate a two-dimensional depiction (Yan and Wu, 2008). This method allowed for a concurrent evaluation of the primary effects of the genotypes and their interactions with the environment. All statistical procedures and biplot visualizations were performed using GenStat statistical software, version 19 (GenStat, 2019), to evaluate genotype performance and environmental stability. The following biplot parameters were utilized:

1. “Which-Won-Where”. Too: An environmental-centered (centering = 2) and G-E scaled (scaling = 0) polygon view was generated. Genotypes located at the vertices of the polygon were identified as the best performers for the environments falling within that specific sector.
2. Mean vs. Stability (Average Environment Coordination - AEC): The “Average Environment View” was used to rank genotypes. The AEC abscissa (single-arrowed line) indicated the mean performance of genotypes across all environments, while the AEC ordinate (perpendicular line) represented stability; genotypes with shorter projections from the abscissa were considered more stable.

#### 2.4.4 Wricke’s ecovalence

Genotypic stability was further assessed using Wricke’s ecovalence (W²) (Equation 3) as described by Wricke (1962), which estimates the contribution of each genotype to the total GEI sum of squares. This statistic measures the deviation of genotype performance from the environmental mean response and is expressed as:

(3) $$W^{2}=\;\sum {\left(X_{ij}-\;{\bar{X}}_{i.}-\;{\bar{X}}_{.j}+\;{\bar{X}}_{..}\right)}^{2}$$

Where Xij is the micronutrient grain of the *ith* genotype in the *j*th environment ${\bar{X}}_{i.}$. ​represents the mean micronutrient gain of the *i*th genotype across all environments; ${\bar{X}}_{.j}$. ​dicates the mean micronutrient grain of the *j*th environment across all genotypes; and refers to the overall mean micronutrient grain of all genotypes across all environments. Genotypes with smaller W² values contribute less to GEI and are therefore considered more stable under varying environmental conditions.

#### 2.4.5 Genotype stability index

Farshadfar (2008) proposed a stability index (Equation 4) based on the combined rank of the genotype mean performance across environments (RY) and the rank of the AMMI Stability Value (RASV).

(4) GSI=RASV+RY

## Results

### 3.1 Analysis of variance and the magnitude of G, E, and G×E effects

The AMMI analysis revealed significant effects of both genotypes and environments for all five micronutrients, indicating that genetic potential and growing conditions jointly influenced grain micronutrient concentrations (**Table 4**). The magnitude of the GEI differed among elements, suggesting that stability was nutrient-specific rather than uniform across traits.

**Table 4:** AMMI analysis of variance for grain micronutrient content in seven wheat genotypes across six environments.

Source of variation	df	Fe	Mn	Zn	Cu	Se
Mean Squares						
Treatments	41	1645.2 ***	348.4 ***	297.9 ***	4.046 ***	643286 ***
Genotypes (G)	6	10573.0 ***	480.0 ***	257.9 **	1.811 ***	1026648 ***
Environments (E)	5	408.8 ***	1978.4 ***	1277.4 ***	28.872 ***	2893986 ***
Block	12	64.2 ns	33.3 ns	74.8 ns	0.232 ns	33315 ns
Interaction (G×E)	30	65.7 ns	50.4 **	142.7 **	0.355 ns	191498 ***
IPCA 1	10	154.8 **	88.6 ***	273.9 ***	0.783 **	395416 ***
IPCA 2	8	34.6 ns	42.7 ns	140.9 *	0.248 ns	163766 *
Residual	12	12.2	23.5	34.6	0.069	40054
Error	72	48.2	23.5	62.2	0.257	62487
% of G×E SS explained						
IPCA 1 + IPCA 2		99.63%	90.59%	78.57%	93.05%	90.16%

***, **, * Significant at the 0.001, 0.01, and 0.05 probability levels, respectively; ns, not significant.

The relative contributions of these components varied across the elements. Environmental effects (E) accounted for the largest proportion of total variation for Mn, Zn, Cu, and Se. In contrast, the genotypic effect (G) was the primary source of variation for Fe content, exceeding both the E and G×E components. The GEI was statistically significant (P< 0.01) for Mn, Zn, and Se but was non-significant for Fe and Cu.

The initial two multiplicative factors (IPCA1 and IPCA2) of the AMMI model accounted for a significant portion of the GEI sum of squares, varying from 78.57% for Zn and 90.16% for Se to 99.63% for Fe (**Table 4**).

### 3.2 Average performance and AMMI stability of genotypes

Mean performance differed among genotypes for each micronutrient (**Table 5**). The Local genotype generally exhibited higher Fe, Mn, and Zn concentrations, whereas IC8 consistently showed elevated Se levels across environments. Other genotypes displayed intermediate performance, often excelling for specific nutrients but not others.

**Table 5:** Mean performance and AMMI Stability Value (ASV) ranking of seven wheat genotypes for five micronutrients.

Genotype	Fe ppm	ASV (Rank)	Mn ppm	ASV (Rank)	Zn ppm	ASV (Rank)	Cu ppm	ASV (Rank)	Se ppb	ASV (Rank)
IC17	45.08	12.04 (6)	34.14	1.55 (4)	46.87	8.93 (7)	**4.94**	1.92 (6)	722.3	24.67 (3)
IC8	**88.62**	**1.05** (1)	30.86	1.42 (3)	42.11	3.45 (2)	4.64	1.57 (5)	**1316.6**	18.00 (2)
Local	**91.27**	3.15 (3)	**44.47**	7.46 (6)	**50.29**	5.67 (6)	4.24	0.66 (2)	827.4	**17.05 (1)**
P3	50.13	4.97 (5)	33.45	**0.59 (1)**	39.21	5.64 (5)	4.52	1.47 (4)	910.7	55.55 (6)
P5	40.32	1.42 (2)	31.13	2.06 (5)	46.83	4.06 (3)	4.63	**0.27 (1)**	710.1	32.45 (5)
Sids12	40.92	22.75 (7)	35.93	1.23 (2)	43.08	**2.13 (1)**	**4.86**	0.91 (3)	579.8	69.42 (7)
YR	87.11	4.61 (4)	41.31	7.75 (7)	42.10	4.61 (4)	4.06	4.05 (7)	727.9	30.63 (4)

Bold values indicate the best performers (highest mean/lowest ASV rank).

Stability indices highlighted differences in genotype responsiveness across environments. IC8 and P5 showed relatively stable performance for Fe, while P3 exhibited lower interaction effects for Mn. Sids12 demonstrated comparatively stable Zn levels, and P5 showed favorable stability for Cu. For Se, IC8 combined a high mean concentration with relatively consistent performance.

**Figure 2:**
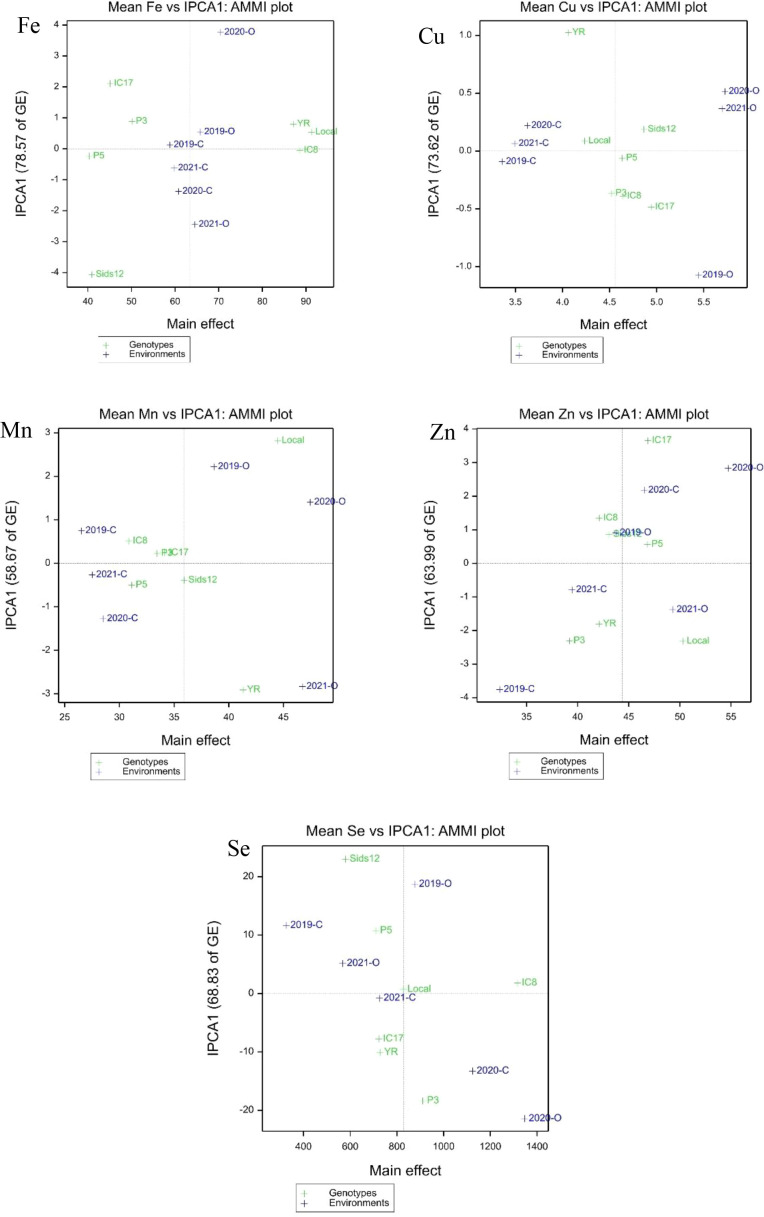
AMMI1 biplots for grain micronutrient concentrations (Fe, Mn, Zn, Cu, and Se) in seven wheat genotypes across six environments. The x-axis represents the main effects (mean concentration), and the y-axis represents the first interaction principal component (IPCA1).

The ranking biplots (e.g., the ranking biplot for Fe (Total – 99.63%), **Figure 2**) offer a visual validation of these findings. In the Fe biplot, the top mean performers, Local and IC8, are located on the right side of the average environment axis (AEA), affirming their exceptional content. The perpendicular distance from the AEA ranks the genotypes based on their average performance, whereas the distance from the biplot origin indicates stability; IC8’s location nearer to the origin than Local corresponds with its superior ASV ranking.

### 3.3 Which-won-where: genotype efficacy by environment

The AMMI model estimates for every genotype-by-environment combination offer an in-depth perspective on adaptation (a portion of this data is shown in **Table 6**). The Local genotype achieved the highest rank in four out of six environments for Fe and Mn. IC8 consistently ranked within the top three performers for Fe across all environments and was the highest-rated genotype for Se in five out of six environments. The GGE biplots featuring convex hulls (e.g., Scatter plot (Total – 90.59% Mn convex) clearly demonstrate these “which-won-where” patterns. The vertex genotypes of the hull signify the top performers in one or several environments. The Mn biplot probably displays Local and YR as vertex genotypes, dominating distinct sectors of the plot.

**Table 6:** Winning genotypes based on AMMI estimates for each nutrient in each environment.

Environment	Fe	Mn	Zn	Cu	Se
2019-C	Local	Local	Local	Sids12	IC8
2019-O	Local	Local	P5	IC17	IC8
2020-C	Local	YR	P5	Sids12	IC8
2020-O	Local	Local	IC17	Sids12	IC8
2021-C	Local	Local	Local	Sids12	IC8
2021-O	Local	YR	Local	IC17	Local

### 3.4 Comprehensive stability assessment using multi-parameter analysis

A thorough multi-parameter stability (**Table 7**) assessment was performed to identify wheat genotypes exhibiting consistent performance and low sensitivity to environmental variations across five micronutrients (Fe, Mn, Zn, Cu, and Se). Distinct variations among genotypes were noted based on the stability metric and nutrient assessed. For iron (Fe), Local and IC8 were consistently ranked highly in terms of superiority, Wricke’s ecovalence, and mean-rank indices, demonstrating that both genotypes show high and stable Fe accumulation in various environments. Likewise, the stability of manganese (Mn) supported Local and YR in terms of superiority and mean ranks, while P3 exhibited the lowest interaction contribution according to Wricke’s ecovalence, indicating it as the most interaction-stable genotype for Mn.

**Table 7:** Top three genotypes based on multiple stability parameters for each nutrient.

Nutrient	Superiority	Static stability	Wricke’s ecovalence	Mean ranks
Fe	1. Local 2. IC8 3. YR	1. IC8 2. P5 3. Local	1. Local 2. IC8 3. P3	1. Local 2. IC8 3. YR
Mn	1. Local 2. YR 3. Sids12	1. IC8 2. P3 3. IC17	1. P3 2. IC8 3. IC17	1. Local 2. YR 3. Sids12
Se	1. IC8 2. P3 3. Local	1. Sids12 2. P5 3. IC8	1. YR 2. IC17 3. P5	1. IC8 2. P3 3. Local
Zn	1. Local 2. IC17 3. P5	1. P5 2. IC8 3. Local	1. Local 2. Sids12 3. IC8	1. Local 2. P5 3. YR
Cu	1. IC17 2. IC8 3. Sids12	1. P5 2. Local 3. Sids12	1. Sids12 2. P5 3. IC17	1. P5 2. Local 3. Sids12

For selenium (Se), IC8 consistently outperformed, ranking among the top three in all stability parameters and securing the first position for mean ranks, validating its strong and extensive adaptability for Se biofortification. Conversely, zinc (Zn) stability trends highlighted the adaptability of Local, which scored highest in terms of superiority, equivalence, and average ranks, validating its effectiveness as a stable Zn accumulator. In the meantime, P5 and IC8 showed robust static stability, reflecting reliable Zn performance across different conditions. For copper (Cu), stability was primarily influenced by P5, which ranked first for static stability and mean ranks and second for Wricke’s ecovalence, highlighting its reliable performance and minimal GEI. IC17 and IC8 also showed elevated superiority values.

The multi-parametric assessment highlights Local as the most stable and widely suited genotype for Fe, Zn, and Mn, whereas IC8 demonstrates superior stability in Fe and Se. In comparison, P5 stands out as the most reliable genotype for Cu.

### 3.5 Radar chart–based multi-index stability assessment of wheat genotypes across environments

**Figure 3:**
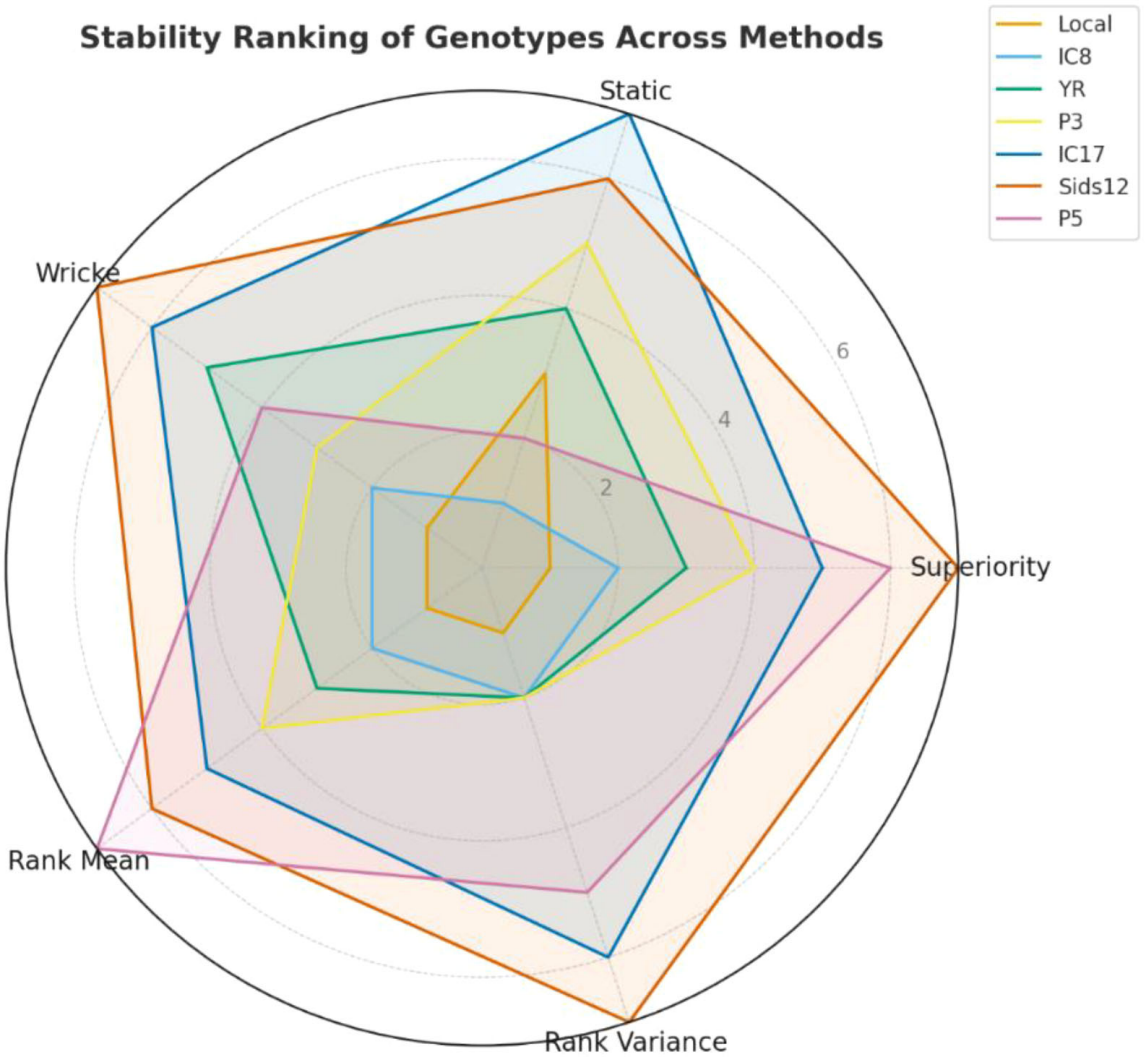
Stability radarchart of seven wheat genotypes (Local, IC8, YR, P3, IC17, Sids12, and P5) across five stability indices (Superiority Index, Static Stability, Wricke’s Ecovalence, Rank Mean, and Rank Variance). Genotypes positioned closer to the center demonstrate greater stability across environments for both grain yield and micronutrient concentrations (Fe, Zn, Mn, Cu, and Se).

The radar chart (**Figure 3**) illustrates the stability rankings of seven wheat genotypes—Local, IC8, YR, P3, IC17, Sids12, and P5—based on five stability statistics (Superiority Index, Static Stability, Wricke’s ecovalence, Rank Mean, and Rank Variance). Since all axes are based on stability, genotypes closer to the center of the chart signify enhanced stability in nutrient levels across different environments.

Throughout the stability indices, Local, IC8, and YR were closely grouped around the center across all indices, signifying robust stability in grain yield and micronutrient content. These three genotypes showed consistently moderate-to-high levels of Fe, Zn, Mn, Cu, and Se, with slight variations across test environments. The tight radial range for these genotypes implies consistent expression of nutrient accumulation pathways and minimal vulnerability to environmental fluctuations. Conversely, Sids12, IC17, and P5 were located at the outer edges of the radar plot, highlighting instability in both agronomic and nutrient-related characteristics. These genotypes demonstrated elevated Wricke’s ecovalence and superiority index scores, indicating significant genotype × environment interaction (GEI), especially concerning Fe and Zn levels. For example, Sids12 demonstrated elevated Zn and Fe levels in certain settings but underperformed in others, leading to diminished overall stability.

P3 held a middle ground, demonstrating moderate stability in both grain production and nutrient characteristics. P3 exhibited fairly stable Zn and Mn levels but demonstrated more variability in Cu and Fe, in contrast to the very stable genotypes.

The overall stability analysis demonstrates distinct clustering of nutrient-stable genotypes (Local, IC8, YR) in contrast to nutrient-unstable genotypes (Sids12, IC17, P5), while P3 acts as a moderate performer.

### 3.6 Micronutrient (Fe, Cu, Mn, Zn, Se) stability based on GGE biplot analysis

The AMMI1 biplots (**Figure 2**) provided a visual integration of mean performance and stability, where the x-axis indicates the main effect (mean) and the y-axis (IPCA1) indicates the magnitude of the G×E interaction. Genotypes or environments positioned closer to the origin (IPCA1 = 0) are considered more stable, while those further away contribute more to the interaction variance. For Fe, genotypes Local, YR, and IC8 were positioned to the right of the grand mean, confirming their superior iron density. Specifically, IC8 was situated closest to the IPCA1 origin, identifying it as the most stable high-Fe genotype. For Cu, Sids12 and IC17 showed the highest mean concentrations, with P5 exhibiting the highest stability near the zero-intercept of the IPCA1 axis. For Mn, the Local genotype exhibited the highest mean concentration but was positioned further from the IPCA1 origin. In contrast, Sids12 and P3 showed higher stability (lower IPCA1 scores) but lower mean concentrations. For Zn, the genotypes Local and IC17 surpassed the grand mean; however, Sids12 and IC8 were identified as the most stable across environments due to their proximity to the IPCA1 origin (IPCA1 = 63.99% of interaction explained). The Se biplot revealed the highest degree of environmental differentiation. Genotype IC8 was the clear outlier for performance, positioned far to the right of the grand mean. The Local genotype demonstrated the highest stability (closest to the origin) for Se content.

**Figure 4:**
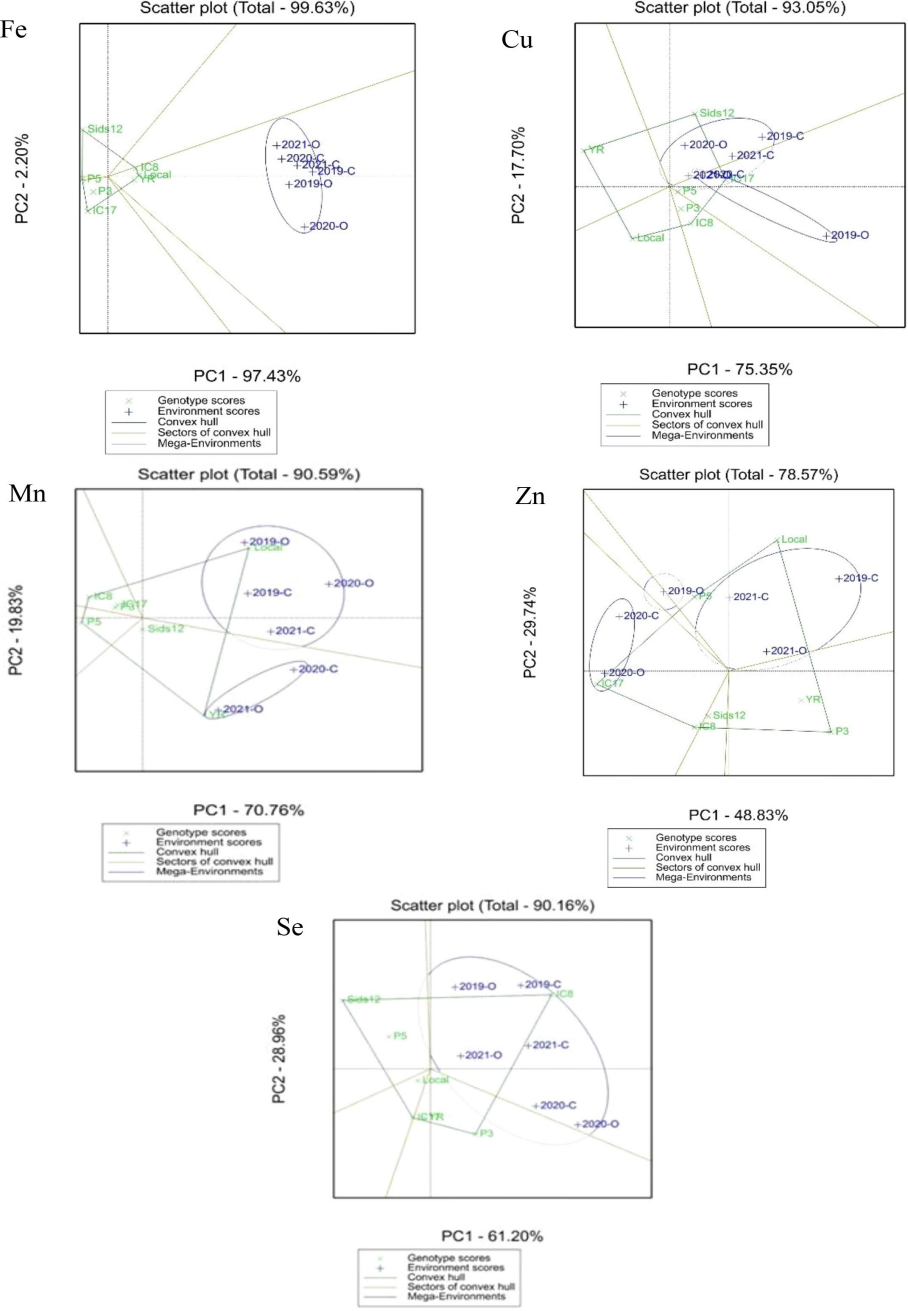
Polygon view of the GGE biplot for micronutrients (Fe, Zn, Mn, Cu, and Se) showing the “which-won-where” among seven wheat genotypes across organic (O) and inorganic fertilization (N) environments.

The polygon representation of the GGE biplot for micronutrient levels (Fe, Cu, Mn, Zn, and Se) offered a detailed illustration of the “which-won-where.” It facilitated accurate identification of genotypes that excelled under environmental conditions (**Figure 4**). The initial two principal components (PC1 and PC2) accounted for a significant share of the overall variation in micronutrient performance across different environments, suggesting that the biplot represented the primary structure of the GEI. In a manner akin to yield-based GGE analysis, the micronutrient biplot divided the test environments into two primary mega-environments, with each linked to different vertex genotypes.

For Fe and Mn, Local, IC8, and YR became prominent vertex genotypes, prevailing under conditions of increased micronutrient availability. These genotypes were consistently located near the polygon vertices and showed robust adaptive responses to environments conducive to mineral absorption. Conversely, P5 and IC17 occupied vertex positions primarily in settings with elevated Cu and Zn expression, indicating that these genotypes have trait-specific physiological mechanisms that facilitate enhanced micronutrient accumulation under specific environmental conditions. For Se, IC8 continued to be the primary vertex genotype, validating its position as the most effective and high-performing genotype for selenium enrichment in both conventional and organic systems.

**Figure 5:**
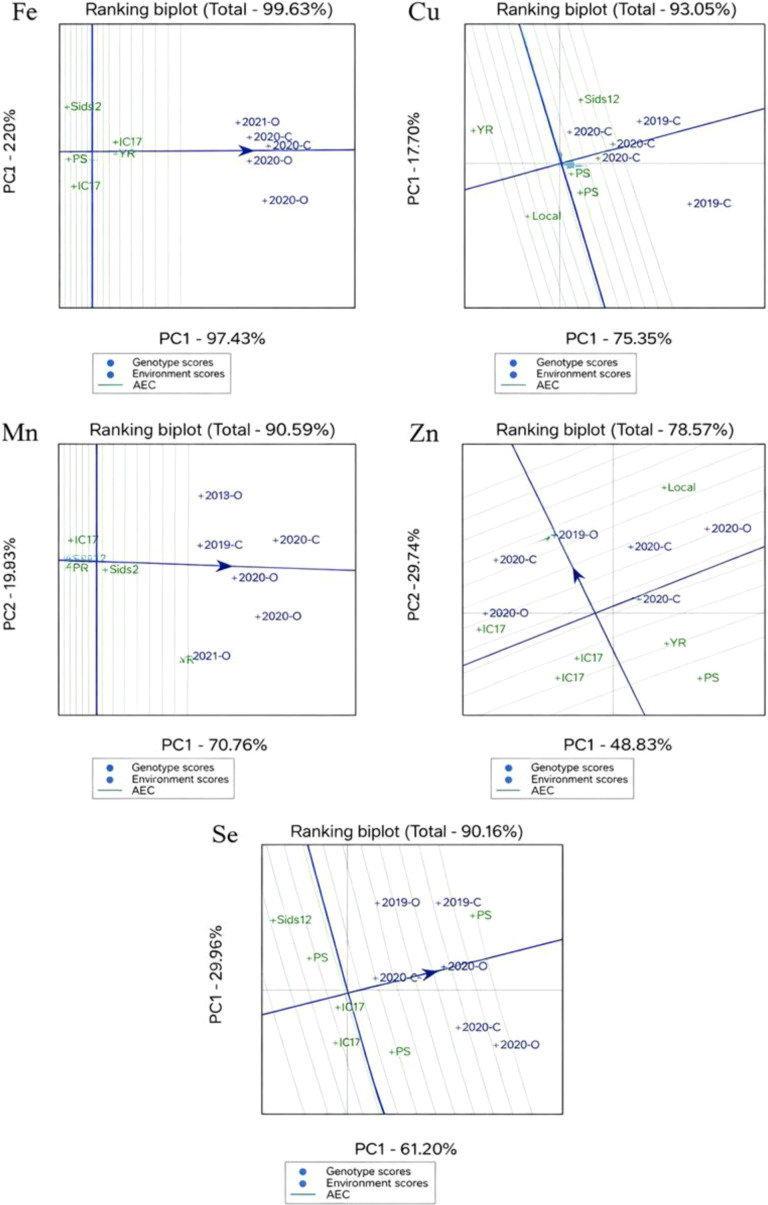
Mean vs. stability view of GGE biplot for micronutrients (Fe, Zn, Mn, Cu, and Se) showing the mean performance and stability of seven elite wheat genotypes in organic (O) and inorganic fertilization (N) environments.

The mean versus stability perspective of the GGE biplot (**Figure 5**) was used to concurrently assess the average performance and consistency of seven top wheat genotypes regarding grain micronutrient levels (Fe, Zn, Mn, Cu, and Se) in both organic (O) and inorganic (N) fertilization settings. The initial two principal components (PC1 and PC2) accounted for a significant share of the overall genotype plus genotype × environment (G + GE) variation. The GGE biplot for iron (Fe) explained 99.63% of the total variation, indicating an excellent fit. Genotypes located closer to the positive side of the average environment coordination (AEC) axis showed higher average Fe concentrations, while those nearer to the AEC origin displayed more stability across different environments. One genotype, Local, achieved the highest rank for average Fe content and showed significant stability, whereas genotypes farther from the origin, such as Sids12 and IC17, were more sensitive to environmental changes and less stable. For copper (Cu), the initial two principal components represented 93.05% of the overall variation. The biplot distinctly differentiated genotypes according to both average Cu concentration and stability. Local and IC8 were situated near the AEC abscissa with brief projection lengths. Conversely, P5 and Sids12 showed extended vectors from the AEC, indicating a greater genotype × environment interaction and lower stability. The GGE biplot accounted for 90.59% of the total variation for manganese (Mn). Genotypes located close to the center of the biplot, especially Local and YR, showed stable Mn performance in both organic and inorganic systems. The zinc (Zn) biplot accounted for 78.57% of the overall G + GE variation, and while this was less than for other micronutrients, it remained adequate for significant interpretation. Genotypes exhibiting greater average Zn concentration were positioned towards the positive side of the AEC abscissa, while stability was deduced from closeness to the AEC ordinate. The Local genotype consistently exhibited a relatively high Zn content alongside satisfactory stability, while P3 and IC17 demonstrated notable instability across different environments. The GGE biplot accounted for 90.16% of the overall variation for selenium (Se). The ranking biplot showed distinct variations among genotypes in terms of mean Se concentration and stability. Local and IC8 were recognized as excellent genotypes, merging elevated Se levels with significant stability in both organic and conventional fertilization. Other genotypes, located further from the AEC origin, showed variable Se accumulation based on the production system.

## 4 Discussion

The significant GEI observed for many micronutrients highlights the intricacies of biofortification breeding. Our findings indicate that micronutrient density is not a fixed characteristic but a flexible reaction arising from the interaction between genetic capability and environmental factors (Naik et al., 2020). Similar patterns have been reported in previous studies examining micronutrient stability in cereals (Saltzman et al., 2013). Although G×E was significant for Mn, Zn, and Se, the lack of significance for Fe and Cu indicates an additive gene effect that facilitates selection across various management strategies.

### 4.1 Implications for organic and traditional fertilization systems

Incorporating fertilization systems into the GGE analysis showed that organic and conventional settings often acted as distinct mega-environments for Zn, Mn, and Se, though this was not the case for Fe. This differentiation has significant consequences for breeding approaches focused on improving nutritional quality. Fe enhancement can depend on widely adapted genotypes assessed across various systems, whereas Zn, Mn, Cu, and Se enrichment necessitates focused selection under nutrient management circumstances to address management-sensitive G×E interactions (Govindan et al., 2022; Govindan and Singh, 2021).

The stability parameters also revealed that Local and IC8 had lower rank variance and Wricke’s ecovalence values, indicating less environmental sensitivity and improved genetic buffering ability. Conversely, Sids12, P5, and IC17 showed significant instability, especially for Fe and Zn, linked to pronounced GEI due to variations in soil nutrients and climate changes (Magallanes-López et al., 2017).

The GGE biplot offered an extensive evaluation of average performance and stability, aiding in the recognition of mega-environments and genotype-specific adaptability in organic and conventional systems (Yan, 2011; Akinwale et al., 2014; Kaya et al., 2021). Incorporating nutrient management practices into stability analysis is crucial for creating successful wheat biofortification initiatives focused on enhancing nutritional security across various production systems.

A critical finding in this study was the predominant influence of the environment (E) on Mn, Zn, Cu, and Se. This suggests that the management system, specifically the contrast between organic fertilization and conventional mineral systems, exerts more selective pressure on these nutrients than the genotype itself. Organic systems often enhance soil microbial activity and chelation, which can increase the bioavailability of Zn and Cu compared to the high solubility, but often fixed mineral fertilizers are used in conventional systems (Burgueño et al., 2008). In our study, the substantial “E” variance indicates that seasonal fluctuations and the slow-release nature of organic nutrients significantly altered the ranking of genotypes for Mn and Se, whereas the genotypic (G) stability of Fe suggests that iron uptake mechanisms (such as phytosiderophore secretion in Strategy II plants) are more genetically buffered against shifts in fertilization regimes (Cakmak and Kutman, 2018; Borg et al., 2012).

### 4.2 Nutrient-specific GGE and stability dynamics

Iron (Fe) showed the most significant genetic regulation among all characteristics. The GGE and AMMI1 biplots highlighted IC8 and the Local genotype as the top performers. The insignificant GEI for Fe is an important breeding finding; it indicates that Fe-rich genotypes retain their dominance irrespective of being cultivated under organic or conventional management. The “Which-Won-Where” model verified Local as a vertex genotype, yet IC8’s closeness to the AEC abscissa (low ASV rank 1) identifies it as the “Ideal Genotype,” merging high average Fe with almost perfect stability. This corresponds with recent research by Govindan et al. (2022), who observed that the heritability of Fe density in wheat typically exceeds that of Zn. In contrast to Fe, Zn was significantly affected by G×E interactions (P<0.01P< 0.01P<0.01). The biplot analysis determined Sids12 to be the most stable (ASV rank 1), even though its average Zn was less than that of the Local genotype. The notable interaction implies that Zn-efficient genotypes might be specific to certain systems; for example, some lines might perform better in organic settings due to improved mycorrhizal connections that aid Zn absorption (Tyagi et al., 2023; Saltzman et al., 2013). The volatility of IC17 for Zn indicates significant sensitivity to variations in soil Zn, rendering it hazardous in uncertain conditions (Saltzman et al., 2013).

The accumulation of Mn was primarily influenced by environmental factors, with the Local genotype reaching the highest average but exhibiting notable variability (ASV rank 6). The GGE biplot revealed that P3 is the genotype with the highest stability for Mn. Within biofortification, although Local presents great promise, its susceptibility to environmental changes is probably a result of Mn significant redox sensitivity in different soil moisture levels, which requires careful consideration (Magallanes-López et al., 2017).

The absence of considerable G×E for Copper (Cu) indicates that, similar to Fe, Cu accumulation remains consistent between organic and conventional systems. P5 was recognized as the most stable genotype. This stability is probably attributed to the inherent nature of Cu transporters in wheat, which seem to be less reactive to the environmental gradients examined here when compared to the highly sensitive Se or Zn (Hassani et al., 2018).

Selenium (Se): Se exhibited the most striking reaction to environmental changes. IC8 excelled, located distinctly to the right on the AEC abscissa, signifying a remarkable genetic ability for Se loading. Despite the notable G×E, IC8 achieved a high stability rank (ASV 2), classifying it as a “dual-purpose” genotype appropriate for both high-input and low-input systems. The pronounced E effect for Se aligns with its reliance on soil pH and redox potential, which vary considerably between organic and conventional management (White, 2016; Schiavon et al., 2017).

### 4.3 Breeding implications

The identification of nutrient-dense and environmentally stable genotypes represents a critical step toward the development of biofortified wheat cultivars adapted to sustainable production systems. The present multi-environment analysis demonstrated that micronutrient accumulation is governed by both genetic effects and GEI, with stability patterns varying among individual elements. This has direct implications for breeding strategy, particularly in the context of simultaneous selection for grain nutritional quality and wide adaptation under contrasting organic and conventional fertilization regimes. The Local genotype exhibited consistently high mean concentrations for Fe, Mn, and Zn across environments, coupled with favorable rankings for superiority and mean stability indices. The recognition of the Local genotype as a leading performer for Fe, Zn, and Mn is an important discovery. Landraces frequently act as sources of lost genetic diversity, evolving through selection in low-input environments that may enhance efficient nutrient uptake and distribution (Garg et al., 2018).

From a breeding perspective, such genotypes represent valuable donor parents for improving Fe and Zn density through conventional or marker-assisted selection pipelines, especially in low-input or organically managed systems where nutrient availability is often constrained. In contrast, IC8 demonstrated superior selenium (Se) accumulation along with moderate-to-high stability across environments, indicating a robust physiological capacity for Se acquisition and retention. The consistent performance of IC8 across both fertilization systems highlights its potential as a parental line for Se biofortification programs targeting broad adaptation. Given the relatively strong GEI observed for Se, the incorporation of IC8 into crossing schemes may enhance buffering capacity against environmental variability in grain Se content. Genotypes such as P3 and P5, which exhibited lower interaction effects for Mn and Cu, respectively, despite moderate mean performance, may serve as stability donors in hybridization programs. The integration of these genotypes into breeding populations could improve nutrient consistency across environments by reducing GEI sensitivity, thereby contributing to the development of nutritionally reliable cultivars (Greveniotis et al., 2023). This approach aligns with breeding objectives aimed at minimizing micronutrient fluctuation under organic and conventional fertilization conditions.

## 5 Conclusion

This study demonstrated that both genetic and environmental factors contribute substantially to variation in wheat grain micronutrient content, with GEI playing a key role across all five elements examined. The results underline the importance of evaluating genotypes across multiple environments when selecting candidates for micronutrient biofortification.

Among the evaluated genotypes, Local showed consistently high concentrations of Fe, Mn, and Zn, while IC8 combined high Se content with relatively stable performance across environments. Other genotypes displayed nutrient-specific advantages, indicating their potential value as parental material in targeted breeding programs. The joint application of AMMI-derived stability statistics and GGE biplot-based mega-environment analysis proved effective for distinguishing genotypes with wide adaptation (e.g., Local for Fe, Mn, and Zn) with specific adaptation to environments or micronutrients. In practical breeding terms, these results indicate that selection indices should weigh both mean micronutrient concentration and stability parameters, rather than relying on average performance alone.

Overall, the identified genotypes—especially Local for Fe/Mn/Zn and IC8 for Se—represent concrete, high-value genetic resources for wheat biofortification programs. Their deployment as parental lines can accelerate the development of cultivars with reliably enhanced grain micronutrient content under both organic and inorganic production systems, particularly when matched to the mega-environments in which they showed the greatest advantage.

## Data Availability

The raw data supporting the conclusions of this article will be made available by the authors, without undue reservation.
